# Factorial Structure Analysis of the Communities that Care Youth Survey (CTC-YS) in Colombian Adolescents

**DOI:** 10.1007/s10935-025-00855-w

**Published:** 2025-06-04

**Authors:** Angela Trujillo, Arcadio de Jesús Cardona-Isaza

**Affiliations:** 1 Facultad de Psicología y Ciencias del Comportamiento, Universidad de La Sabana, Autopista Norte de Bogotá. Chía, Campus del Puente del Común, Km. 7, Chía, Cundinamarca Colombia; 2 Faculty of Psychology, Universidad del Valle, Cali, Colombia

**Keywords:** Communities that care youth survey, Adaptation, Psychometric, Reliability, Risk factors, Protective factors

## Abstract

The “Communities That Care Youth Survey” (CTC-YS) emerges from the Social Development Model and provides a comprehensive assessment of risks and protective factors in community, family, school, as well as individual and peer group domains. This has contributed to the prevention of drug use and other associated factors. The main objective of this study was to analyze the factorial structure of the CTC-YS in Colombian adolescents. A total of 2,963 volunteers between 12 and 19 years participated (mean = 14.25, SD = 1.53; 53% female). Construct validity was assessed through a confirmatory factor analysis of domains. Goodness-of-fit indices were found to be adequate for risk and protective factors in the community and school domains as well as for protective factors in the family and individual and peer group domains. However, the family, individual, and peer group domains did not reach the optimal indices for risk factors, prompting recommendations for potential improvements. Overall, the results support the reliability and construct validity of the survey domains, advocating its utilization in researching and assessing risk and protective factors among Colombian adolescents. The need for additional psychometric analyses was discussed to enhance the validity and applicability of the CTC-YS in the Colombian context.

## Introduction

Assessing risk and protective factors is essential for understanding and addressing adolescent behavioral issues, thereby promoting healthy development and preventing long-term problems (Oesterle et al., 2018). By identifying the factors that create a supportive environment, policies and practices can be enhanced to foster positive adolescent development (Kim et al., 2015).

The Social Development Model (SDM) is a key framework for prevention and intervention, detailing the risk and protective factors for antisocial and prosocial behaviors in adolescents (Catalano & Hawkins, 1996). This model has led to the development of Communities That Care (CTC), a community-based strategy to reduce substance abuse, delinquency, and antisocial behavior among adolescents (Brown et al., 2007). The CTC model promotes intersectoral collaboration and employs a science-based prevention approach to mitigate the impact of risk factors (Kim et al., 2015; Oesterle et al., 2018).

The Communities That Care Youth Survey (CTC-YS), based on the Social Development Model (SDM), is a validated self-report instrument designed to measure an array of risk and protective factors across multiple ecological domains, including community, school, family, peer and individual, alongside adolescent problem behaviors. The authors defined risk factors as characteristics that increase the likelihood of problems behaviors like substance use or delinquency. Protective factors were defined as characteristics that mitigate these risks, either directly or indirectly (Arthur et al., 2002). This instrument was developed to facilitate comprehensive prevention needs assessments and strategic planning for interventions targeting substance use, delinquency, and other problem behaviors. The questionnaire is based on the Communities That Care prevention planning framework, which integrates findings from longitudinal research to identify risk and protective factors predictive of adolescent problem behaviors (Haggerty & Shapiro, 2013; Kuklinski et al., 2021; Oesterle et al., 2018). Factors included in the survey were chosen based on empirical evidence of their predictive validity and were organized into distinct domains: (a) community domain, that measures factors such as low neighborhood attachment and community disorganization; (b) school domain, that includes academic failure and low commitment to school; (c) family domain that assess factors such as poor family management and family conflict, and (d) peer/individual domain, that captures constructs like peer drug use and sensation seeking. Appropriate assessment tools enable the measurement, baseline establishment, and monitoring of these factors (Thurow et al., 2021). Given its importance in evaluating these factors and in prevention programs, this study aimed to analyze the factorial structure of the CTC-YS in Colombian adolescents through confirmatory factor analysis (CFA) for each domain.

### Adaptations of the Communities that Care Youth Survey (CTC-YS)

Initial psychometric studies on the CTC-YS, conducted with a North American population, assessed its development, factor consistency, reliability, and expected relationship between risk and protective factors. The original survey included 121 items to measure the 29 constructs related to these factors (Arthur et al., 2002). CFAs showed adequate goodness-of-fit indices and invariance across sex, education level, and ethnic and racial groups (Glaser et al., 2005). Cutoff points have been established to differentiate adolescents at higher and lower risks of problematic behaviors (Arthur et al., 2007; Briney et al., 2012).

The utility of the CTC-YS for prevention has been demonstrated in diverse populations. Guttmannova et al. (2017) compared a national sample of Native American youth and found good reliability for both variables. The CTC-YS has also assessed risk and protective factors in non-normative groups, such as foster care adolescents (Brook et al., 2015).

Principal component analyses and predictive validity studies support the strength of SDM in explaining these factors (Corrigan, 2014a, b). Additionally, the CTC-YS factors predicted mental health outcomes, as shown by Schwendemann et al. (2018), who used family protective factors to evaluate their impact on depressive symptoms.

A study on South African adolescents using the CTC-YS reported reliability indices for twenty-seven factors, ranging from moderate to high (α = 0.60 to 0.94), and demonstrated that each domain predicted tobacco, alcohol, and marijuana use (Morojele et al., 2002). In Trinidad and Tobago, CFA of the community domain showed adequate fit indices after removing the transition and mobility factors and combining the perceived availability of drugs and weapons factors (Maguire et al., 2011). In Australia, a CFA of the family domain also showed adequate fit indices but required item elimination and factor reduction (Kuttler et al., 2015).

In Malaysia, CTC-YS adaptation involves an EFA with twenty-seven factors (Razali & Kliewer, 2015). Subscale reliability had medium-to-high Cronbach’s alpha values (α = 0.63 to 0.97), excluding the transition and mobility factors and social skills due to low reliability. The Iranian CTC-YS adaptation uses CFAs for twenty-nine factors, yielding good reliability and fit indices (Baheiraei et al., 2016). In Brazil, a pilot study of twenty-five risk factors showed low reliability for school failure and rebelliousness (De Oliveira Corrêa et al., 2022).

Recently, risk and protective factors have been measured in several European countries, using representative samples. The CTC-YS reliability varied by country, with higher indices in Germany, the Netherlands, and Croatia (Farrington et al., 2021). A recent pilot study on CTC-YS adaptation in Estonia was published (Siilbek & Streimann, 2024).

The psychometric analysis of the CTC-YS for Colombia is necessary because of its relevance in assessing risk and protective factors. It has not yet been validated in the local context, despite the history of implementation in the country of programs based on the SDM and using the CTC-YS as an assessment instrument (Mejía-Trujillo et al., 2015). Furthermore, it is standard practice for all instruments to be adapted and validated for specific contexts, ensuring validity criteria that detect cultural differences, adapt the local language, and guarantee data reliability (Fenn et al., 2020). Proper adaptation optimizes economic and human resources by avoiding generic interventions and ensures that preventive actions adequately address the population’s needs. Additionally, prior adaptations of the CTC-YS in other countries have shown significant variations (Baheiraei et al., 2016; Kuttler et al., 2015; Maguire et al., 2011; Siilbek & Streimann, 2024).

In Colombia, longstanding contextual problems have persisted for decades and must be considered when assessing the risk factors affecting adolescents. These issues include social exclusion, lack of opportunities, poverty, limited access to educational and cultural services, forced displacement, and exposure to crime and violence (Alvarado et al., 2021). Although the CTC-YS assesses many of these factors, it is possible that some items in the original instrument do not adequately represent the reality of Colombian adolescents. For example, mobility in Colombia is often associated with forced displacement and poverty rather than simple residential changes. Similarly, instead of gang affiliation, adolescents are more commonly exposed to recruitment by guerrillas, organized criminal groups, or forced conscription (Alvarado et al., 2021; Esparza et al., 2020).

In Colombia, a study detailed the implementation and adaptation of the “Communities That Care” preventive programs, reporting the reliability of 11 risk factors and three protective factors of the CTC-YS, with Cronbach’s alpha values ranging from 0.57 to 0.91 (Mejía-Trujillo et al., 2015). Other studies in countries with more factors have shown good reliability (Obando et al., 2014). Thurow et al. (2021) noted that researchers have used various analyses to develop the construct validity of the CTC-YS, including item relocation, modification, deletion, inversion, factor grouping, and exclusion (Baheiraei et al., 2016; De Oliveira Corrêa et al., 2022; Glaser et al., 2005; Kuttler et al., 2015; Maguire et al., 2011). These analyses support the factorial structure of the CTC-YS and the validity of the SDM, making the validations diverse. This study analyzed the factorial structure of the CTC-YS based on the Communities That Care Youth Survey Scale Dictionary (Social Development Research Group, 2014).

## Methods

### Participants

The study included 2935 adolescents aged 12 to 19 years (Mean = 14.25, SD = 1.53), with 53% female (*n* = 1558) and that were enrolled in public educational institutions within five selected municipalities within the Department of Cundinamarca, Colombia. These were chosen to reflect a diverse mix of urban and rural settings, with varying levels of access to health and education resources. Two of the municipalities are predominantly urban, characterized by high population density and greater access to infrastructure. The remaining three are rural, with dispersed populations and limited access to services. The majority of participants came from low-to-middle-income households, reflecting the socio-economic profile of public school students in the region. Most participants reported living in two-parent households, with a notable proportion from single-parent families. The distribution of adolescents across grades was as follows: 8% in sixth grade, 21% in seventh grade, 27% in eighth grade, 26% in ninth grade, and 18% in tenth and eleventh grades. Public educational institutions are the primary providers of schooling in these areas. In this study, a sample size calculation was performed to ensure the robustness of the CFA of the CTC-YS using the sample size calculator for structural equation models (SEM) from Analytics Calculators. The model considered 29 latent and 153 observed variables, with a strict significance level (*p* =.01), an expected effect size of 0.3, and a desired statistical power of 0.9. The calculation indicated a minimum sample size of 340 participants, suggesting that the obtained sample exceeds the required minimum (Mundfrom et al., 2005) (Table 1).

**Table 1:** Sample characteristics

Municipality	School	Female	Male	Total	Percentage
Municipality 01	School 01	25	26	51	27.3
	School 02	10	14	24	
	School 03	36	23	59	
	School 04	23	26	49	
	School 05	35	39	74	
	School 06	66	50	116	
	School 07	32	32	64	
	School 08	117	98	215	
	School 09	9	11	20	
	School 10	78	58	136	
Municipality 02	School 01	54	48	102	14.1
	School 02	66	46	112	
	School 03	44	22	66	
	School 04	56	36	92	
	School 05	23	24	47	
Municipality 03	School 01	30	23	53	16.5
	School 02	242	194	436	
Municipality 04	School	199	210	409	13.9
Municipality 05	School 01	11	14	25	28.2
	School 02	21	10	31	
	School 03	60	59	119	
	School 04	113	118	231	
	School 05	26	19	45	
	School 06	16	14	30	
	School 07	72	64	136	
	School 08	17	14	31	
	School 09	64	71	135	
	School 10	13	14	27	
	**TOTAL**	1558	1377	2935	100

### Instruments

The Communities That Care Youth Survey (CTC-YS) (Arthur et al., 2002), comprises 153 items, distributed across 29 scales representing 21 risk factors and 11 protective factors. The structure allows for efficient administration within a 50-minute school period. Each scale typically includes three to five items, score on Likert-type response options to assess agreement or frequency of behaviors. For example, for risk factors, items include questions such as “Do your parents know where you are and who you are with most of the time?”. For protective factors, sample items include, “How close do you feel to your parents?” It also evaluates behavioral outcomes such as drug use and antisocial or criminal behaviors (Rhew et al., 2016). The survey is appropriate for adolescents and features mixed-format questions (polytomous and dichotomous). This study retained the factors and scale items’ acronyms and names from the original, following the Communities That Care Youth Survey Scale Dictionary (Social Development Research Group, 2014). Examples of items for each factor are provided in Supplementary Information SI1. We used the Spanish version of the CTC-YS provided by the original authors and made several adaptations to ensure the survey was culturally relevant and appropriate for the Colombian context: (1) The items about academic grades were adapted to reflect the grading system used in Colombia, which differs from systems in other countries. This ensures that the questions are relevant and understandable for Colombian students. (2) Items about alcohol consumption were updated to include beverages typical in Colombia, such as aguardiente, to accurately capture consumption patterns and habits within the local context. (3) Certain terms were adjusted to match Colombian local terminology. For example: The word “*Escuela*” was replaced with “*colegio”*, as this term is more commonly used to refer to schools in Colombia. (4) Items related to race and ethnicity were excluded because these are not culturally salient or relevant factors in the Colombian context, unlike in other regions where the survey may be applied. (5) The item about school clubs was modified to reflect the reality of extracurricular activities and committees, which are more common in Colombian schools than formalized school clubs.

The CTC-YS aligns with our study objective by providing a robust framework for identifying specific risk and protective factors associated with adolescent outcomes. Using this questionnaire will ensure that the factors measured were not only theoretically grounded but also empirically validated, allowing for precise assessments tailored to Colombian youth.

### Procedure

This study was approved by the Ethics Committee of Universidad de La Sabana, Colombia (REF: 190/22-03-2023) and adhered to the guidelines set forth in the Declaration of Helsinki for research involving human subjects (World Medical Association, 2013).

The research project was formally presented to the health and education departments of each municipality, outlining its objectives, scope, and potential benefits to encourage their participation. Public educational institutions were deliberately selected as key partners to support and facilitate the data collection process. Municipalities that agree to participate were asked to nominate professionals (e.g., school psychologists or social workers) to assist in the study. To ensure standardized data collection procedures, training sessions were organized for the professionals nominated by the municipalities. These sessions were conducted by the principal investigator during 2021–2023 and included a detailed explanation of the study’s objectives and ethical considerations, instructions on administering the research instrument, focusing on maintaining consistency and minimizing potential biases. Informed consent forms were sent to the participants’ legal guardians and collected prior to the data collection sessions. These sessions took place during school hours, in computer labs designated by the participating schools since the questionnaire was applied via Google Forms. At the beginning of each session students were informed of their right to stop answering if they were uncomfortable. In accordance with Colombian ethical standards for psychological studies and the protocol approved by the ethics committee that reviewed the research, the questionnaire was administered by licensed psychologists or social workers with valid professional credentials. This precautionary measure was taken to minimize risks and provide immediate support to adolescent participants in case they experienced any emotional distress.

### Data Analysis

First, a descriptive analysis of study variables, including mean, standard deviation, and reliability (via Cronbach’s alpha and McDonald’s Omega), was conducted (Table 2). Correlation analyses within each domain were performed using SPSS v.26 (IBM, 2019). The factorial validity of the CTS-YS was examined through CFAs for each domain using Mplus 8.10 (Muthén & Muthén, 2017), employing the MLM method for non-normally distributed data, ensuring robust analyses with Satorra-Bentler correction. Fit indices included the Comparative Fit Index (CFI), Tucker-Lewis Index (TLI), root mean square error of approximation (RMSEA), and Standardized Root Mean Square Residual (SRMR). Guidelines for acceptable model fit are: CFI and TLI > 0.90, RMSEA < 0.08 (with 0.05 being good, 0.08 acceptable, 0.10 poor), and SRMR < 0.08 (Hu & Bentler, 1999). Factor loadings should be of sensible magnitude, with absolute values > 0.30 (Tabachnick & Fidell, 2013).

## Results

The results are as follows. Table 2 presents the descriptive and reliability results. Supplementary Information SI2 presents the correlations between the risk and protective factors for each domain. Supplementary Information SI3 shows the CFAs for each domain. Table 3 presents the results of the CFAs for each domain, differentiated by protective and risk factors. All participants who agreed to complete the survey did so fully, as the system (Google Forms) required every question to be answered before submission. Consequently, there were no partially completed surveys. However, approximately 15 to 20 students per school opted not to participate despite having signed informed consent from their legal guardians. These students were not included in the sample.

**Table 2:** Descriptive statistics and reliability of the CTC-YS factors (*n* = 2963)

Variable	Items	M	SD	α	ω
*Community Domain*					
1. Low attachment to the neighborhood	4	9.37	2.20	0.78	0.79
2. Community disorganization	3	12.28	3.17	0.64	0.67
3. Perception of drug availability	4	7.70	3.07	0.77	0.78
4. Perception of weapon availability	2	3.63	1.87	0.91	0.92
5. Laws and norms favorable to drug use	6	10.80	2.76	0.73	0.76
6. Opportunities for prosocial involvement	3	8.04	2.76	0.62	0.63
7. Rewards for prosocial involvement	3	8.54	2.29	0.79	0.79
*Family Domain*					
8. Family History of Antisocial Behavior	9	15.45	6.21	0.69	0.67
9. Poor Family Management	8	14.11	2.36	0.85	0.85
10. Family Conflict	3	5.99	2.00	0.72	0.73
11. Parental Attitudes Favorable to Drug Use	3	3.98	1.30	0.50	0.55
12. Parental Attitudes Favorable to Antisocial Behavior	3	3.75	1.17	0.62	0.62
13. Family Attachment	4	10.50	2.77	0.31	0.28
14. Family Opportunities for Prosocial Involvement	3	6.09	3.48	0.63	0.72
15. Family Rewards for Prosocial Involvement	4	10.74	2.51	0.42	0.52
16. Family History of Antisocial Behavior	9	15.45	6.21	0.69	0.67
*School Domain*					
17. Academic Failure	2	5.02	1.33	0.72	0.72
18. Low School Engagement	7	13.43	3.80	0.63	0.64
19. Opportunities for Prosocial Involvement	5	15.11	2.78	0.63	0.63
20. Rewards for Prosocial Involvement	4	11.24	2.07	0.65	0.66
*Individual and Peer Group Domain*					
21. Rebellion	3	5.67	1.98	0.60	0.61
22. Sensation Seeking	3	6.00	3.09	0.63	0.66
23. Risk Perception of Drug Use	6	10.87	4.58	0.88	0.88
24. Early Initiation of Drug Use	6	6.43	7.39	0.69	0.73
25. Early Initiation of Antisocial Behavior	4	1.38	3.47	0.45	0.48
26. Favorable Attitudes Towards Drug Use	5	8.13	2.74	0.71	0.73
27. Favorable Attitudes Towards Antisocial Behavior	5	8.03	3.07	0.82	0.83
28. Rewards for Antisocial Involvement	4	5.67	2.53	0.80	0.80
29. Friends Who Use Drugs	4	1.79	2.81	0.70	0.72
30. Interaction with Antisocial Peers	7	1.17	2.30	0.70	0.71
31. Intention to Use Drugs	3	5.23	1.68	0.63	0.65
32. Interaction with Prosocial Peers	3	7.35	3.09	0.61	0.62
33. Belief in Moral Order	4	11.66	2.61	0.51	0.51
34. Prosocial Involvement	3	5.20	2.57	0.52	0.52
35. Rewards for Prosocial Involvement	4	12.21	3.81	0.63	0.63

Note M = Mean, SD = Standard Deviation; α = Cronbach’s alpha; ω = McDonald’s omega

**Table 3:** Confirmatory factor analysis (CFA) results by domain and risk and protection factors (MLM Estimator)

	Community domain			Family domain			School domain			Individual and peer group domain		
	C	*R*	*P*	C	*R*	*P*	C	*R*	*P*	C	*R*	*P*
*X* ^*2*^	1843.09	1292.90	37.68	6199.50	2063.37	270.37	7692.14	265.15	214.38	11613.39	8091.67	392.91
*gl*	252	140	7	592	215	39	156	24	23	1667	1112	70
*p*	> 0.001	> 0.001	> 0.001	> 0.001	> 0.001	> 0.001	> 0.001	> 0.001	> 0.001	> 0.001	> 0.001	> 0.001
CFI	928	0.931	0.993	0.811	0.877	0.961	0.919	0.934	0.947	0.757	0.785	0.969
TLI	0.915	0.915	0.985	0.781	0.855	0.945	0.903	0.901	0.918	0.733	0.764	0.960
RMSEAA	0.046	0.053	0.038	0.057	0.055	0.045	0.040	0.058	0.053	0.045	0.046	0.039
CI	044 0.048	0.050 0.055	0.027 0.051	0.055 0.058	0.052 0.056	0.040 0.050	0.037 0.043	0.052 0.065	0.047 0.060	0.044 0.046	0.045 0.047	0.039 0.043
SRMRR	0.049	0.054	0.024	0.079	0.079	0.039	0.037	0.044	0.040	0.082	0.088	0.033

Note X^2^ = Chi-square; *df* = degrees of freedom; CFI = Comparative Fit Index; TLI = Tucker-Lewis Index; RMSEA = Root Mean Square Error of Approximation; SRMR = Standardized Root Mean Square Residual. CI = confidence interval; C = Complete domain model; R = Risk factor model of the domain; P = Protection factor model of the domain

### Community Domain

The Community domain comprises five risk factors and two protective factors. The factor “Perception of availability of weapons” had only one item in the original questionnaire version. Still, we included a new item: “If you wanted to get a firearm, how difficult would it be to get one in your neighborhood?” The reliability of these two items was high (α = 0.91). When conducting the CFA of the community domain, the results of the proposed original model did not demonstrate adequate goodness-of-fit indices (*X*^2^ = 6852.63, *df* = 254, *p* >.001, CFI = 0.703, TLI = 0.649, RMSEA = 0.094 (CI = 0.092 − 0.096), SRMR = 0.08). High covariance was observed between two items, “I feel safe in my neighborhood” and “I would like to move from my neighborhood.” However, the model did not converge upon removal. Therefore, we decided to integrate the first item into the low neighborhood attachment factor. The resulting final equation exhibited satisfactory indices (*X*^2^ = 1843.09, *df* = 252, *p* >.001, CFI = 0.928, TLI = 0.915, RMSEA = 0.046 (0.044 0.048), SRMR = 0.049).

### Family Domain

The family domain comprises five risk factors and three protective factors. The results of the proposed model were unsatisfactory (*X*^2^ = 3334.42, *df* = 427, *p* >.001, CFI = 0.953, TLI = 0.923, RMSEA 0.047 (CI = 0.046 0.049), SRMR = 0.030). In the model, the item “Has any of your siblings ever smoked a cigarette?” from the family history of the antisocial behavior factor did not contribute factorially to any factor in the model. The items from the family history of antisocial behavior factor were distributed into three groups, differentiating substance use by siblings, substance use by adults, and antisocial behaviors of siblings and adults. Items from the poor family management factor distributed loadings into two different factors. Another peculiarity is that the items “I enjoy spending time with my mom” and “I enjoy spending time with my dad,” which belong to the rewards for prosocial family involvement factor, contributed negatively to the factor, and in the case of the item “I enjoy spending time with my mom,” it contributed to the variance of the family attachment factor.

We observed that the domain could have multiple solutions. The model for the protective factors was satisfactory (*X*^2^ = 270.37, *df* = 39, *p* >.001, CFI = 0.961, TLI = 0.945, RMSEA 0.045 (CI = 0.040 0.050), SRMR = 0.039), whereas the model for the risk factors showed moderately good fit indices (X^2^ = 2063.37, *df* = 215, *p* >.001, CFI = 0.877, TLI = 0.855, RMSEA 0.054 (CI = 0.049 0.056), SRMR = 0.079). Because of the inconsistent results observed, it was considered that although the complete original model did not achieve optimal goodness-of-fit indices when integrating family risk and protective factors, the domain is reliable and has a consistent structure with the base model. We decided to retain the original structure with some adjustments to the covariance. The observed goodness-of-fit indices were as follows (*X*^2^ = 6199.50, *df* = 592, *p* >.001, CFI = 0.811, TLI = 0.781, RMSEA 0.057 (CI = 0.055 0.058), SRMR = 0.079).

### School Domain

The school domain comprises two protective factors and two risk factors. The results of the originally proposed model displayed moderate fit indices (*X*^2^ = 1154.699, *df* = 126, *p* >.001, CFI = 0.864, TLI = 0.839, RMSEA 0.050 (CI = 0.049 0.055), SRMR = 0.052). In response, modifications were made by correlating the items “How often during this year did you try to do your best work?” and “How often during this year did you not like being at school?” as well as between the items “How often during this year did you not like being at school?” and “How often during this year did you enjoy being at school?” These modifications belonged to the subscale of low school engagement. After implementing these modifications, the model exhibited satisfactory fit indices (Table 3).

### Individual and Peer Domain

The analyzed individual and peer group domains comprised eleven risk factors and four protective factors. Due to differences with the original test context, the gang participation factor was omitted, and the social skills factor was excluded due to low reliability. The proposed model did not display satisfactory goodness-of-fit indices (*X*^2^ = 16015.55, *df* = 1847, *p* >.001, CFI = 0.670, TLI = 0.639, RMSEA = 0.051 (CI 0.050 0.052), SRMR = 0.079). Further analysis was conducted separately on the 11 risk factors, the results indicated unsatisfactory goodness-of-fit indices (*X*^2^ = 8091.67, *df* = 1112, *p* >.001, CFI = 0.785, TLI = 0.764, RMSEA = 0.046 (CI 0.045 0.047), SRMR = 0.088). Conversely, the model that included only the protective factors exhibited adequate goodness-of-fit indices (*X*^2^ = 392.91, *df* = 70, *p* >.001, CFI = 0.969, TLI = 0.960, RMSEA = 0.039 (CI 0.039 0.043), SRMR = 0.033).

In summary, it was observed that the family and individual and peer group domains might present various factorial solutions that could be satisfactory. There was also significant covariance between some items, and some items contributed to the variance of factors to which they should theoretically not belong, particularly in the protective factors of the family and individual and peer group domains when evaluating the complete domain. This suggests a complex interplay within these domains, which may require further exploration to fully understand the dynamics and interactions among these factors.

## Discussion

The CTC-YS is an instrument based on the Social Development Model (Catalano & Hawkins, 1996), with substantial evidence supporting its reliability and validity across diverse populations. While the survey has been widely applied internationally (De Oliveira Corrêa et al., 2022; Guttmannova et al.; Kuttler et al., 2015; Maguire et al., 2011; Morojele et al., 2002; Razali & Kliewer, 2015) this study is the first to analyze its factorial structure in a representative sample of Colombian adolescents. This contextual specificity is crucial due to Colombia’s unique sociocultural and sociopolitical landscape, which differs significantly from the contexts where the instrument was originally developed and validated. In Colombia, adolescents are exposed to a complex interplay of risk factors, including poverty, violence, forced displacement, and familial instability, which may influence how they interpret survey items and respond to them (Esparza et al., 2020; Marroquín Rivera et al., 2020; Tamayo-Aguledo et al., 2022). For instance, factors like “transition and mobility” in the community domain may not directly align with typical neighborhood-level transitions observed in Western contexts but rather reflect broader systemic issues such as land dispossession or internal displacement due to armed conflict (Esparza et al., 2020). These contextual nuances underscore the necessity of adapting and validating the CTC-YS to ensure its psychometric robustness and practical applicability in Colombian settings. Overall, our results indicate that the instrument has a factorial structure that fits with the original instrument (Arthur et al., 2002). However, there are aspects that need to be discussed regarding the reliability and goodness-of-fit indices of the domains.

It is important to note that psychometric analyses of the CTC-YS differ across contexts in which adaptations have been made. According to Thurow et al. (2021), psychometric analyses of the survey mainly include construct and criterion validity, as well as reliability. Some studies have also provided different validity criteria, but most of these analyses have been conducted with North American populations. A systematic review by Thurow and colleagues concluded that cultural adaptations show satisfactory results but are not as good as those presented in the original. Moreover, CFAs are scarce and generally do not include all factors; in some cases, only one dimension is included, with adjustments and modifications. However, these results are promising and indicate that it is possible to adapt the survey to different cultures (Baheiraei et al., 2016; De Oliveira Corrêa et al., 2022; Kuttler et al., 2015; Maguire et al., 2011).

Regarding the community domain, the reliability of the subscale was adequate (α = 0.62 to 0.91). As indicated, the validations of the CTC-YS varied in the subscales analyzed. For example, in the CFA by Glaser et al. (2005), the subscale ‘opportunities for prosocial involvement’ was omitted, but it has been included in subsequent psychometric analyses (Baheiraei et al., 2016; Maguire et al., 2011). Maguire et al. (2011) conducted a CFA that only considered the community domain, which consisted of two protective factors and four risk factors, excluding “transition and mobility” and “low neighborhood attachment.” Our results show that the version of the CTC-YS we analyzed presents a factorial structure that aligns with the proposed model (Social Development Research Group, 2014). The “transition and mobility” factors were excluded due to its limited relevance to Colombian adolescents as operationalized in the original survey. In Colombia, this factor may better reflect displacement due to violence or economic necessity rather than voluntary movement. Additionally, the strong covariance between items related to safety and neighborhood attachment highlights the role of crime and insecurity in shaping adolescent perceptions, further emphasizing the need for cultural adaptation of this domain (Esparza et al., 2020).

In the different solutions analyzed in the family domain, some items from the protective factors shared variance with items in the risk factors. Analyzing the possible reasons, this could be due to the participants’ different interpretations of the items, which originate in contextual differences and the social roles of the parents. The real conditions in the country can influence the configuration of family relationships. For instance, Colombia has markedly sexist (machista) traditions and ideas, which impact education and family dynamics and are associated with a high rate of household abandonment (Vigoya, 2011). Furthermore, a significant emotional distance from fathers is observed among the study participants: 76.4% stated that they do not enjoy spending time with their fathers, while this percentage was only 3.5% in the case of their mothers. Similarly, 23.6% reported not feeling close to their father, compared to 17.6% who expressed the same regarding their mother. This discrepancy reflects broader societal issues, such as the machismo culture, that shape family dynamics and may result in variances in how risk and protective factors are perceived and measured (Vigoya, 2011). These contextual differences may explain why some items showed shared variance between protective and risk factors, suggesting the need for item rewording or separation (e.g., distinct scales for maternal and paternal relationships).

Adjustments and modifications to the models have been made in previous studies on CFAs in the family domain. For example, Glaser et al. (2005) subdivided the attachment subscale into attachment to the father and attachment to the mother. Baheiraei et al. (2016) transferred values between factors. Additionally, Kuttler et al. (2015) analyzed only the family domain without the antisocial behavior family history subscale, resulting in a reduction with adequate goodness-of-fit indices for a model with five subscales. Conversely, the pilot study by De Oliveira Corrêa et al. (2022) included only the risk factors of the domain. Regarding this domain, we consider that before suggesting the elimination of items, the wording of adapted items should be analyzed, and verification processes, such as test-retest and the use of multiple samples, should be employed.

The results for the school domain were satisfactory, retaining the two protective and risk factors. These results were also observed in other CFAs, for example, in Baheiraei et al. (2016) and Glaser et al. (2005), where modification of indices was required in the low school commitment subscale. Regarding reliability, the values were acceptable (α = 0.63 to 0.72), which aligns with the average observed in other studies (Brown et al., 2009; De Oliveira Corrêa et al., 2022).

In the individual and peer group domains, adequate goodness-of-fit indices were observed for the protective factors. Other CFAs of the individual and peer group domains were conducted using a different number of factors. The study by Glaser et al. (2005) included ten factors; Baheiraei et al. (2016) analyzed 14 factors, and the pilot study by De Oliveira Corrêa et al. (2022) had indices in the CFA for nine risk factors. None of the previous CFAs included protective factors related to participation and rewards for prosocial involvement, making it difficult to determine whether the contradictory associations of prosocial participation with risk factors observed in this study occurred in other CFAs.

Possible explanations for these contradictory associations may lie in the cultural and contextual factors unique to Colombian adolescents. Prosocial participation, often linked to positive developmental outcomes, may intersect with certain risk factors due to overlapping contexts where these behaviors occur. For instance, in some communities, activities traditionally viewed as prosocial, such as participation in neighborhood or peer-group activities, might occur in environments where exposure to risky behaviors (e.g., substance use or delinquency) is prevalent. Adolescents may engage in prosocial behaviors but remain influenced by their immediate context, blurring the boundaries between protective and risk factors. Also, peer influence plays a significant role in adolescent behavior. In some cases, prosocial activities may be driven by peer networks that simultaneously expose adolescents to risk. For example, participation in sports or local community events might also involve peer-led behaviors associated with risk-taking.

Understanding these contradictory associations requires acknowledging the complex interplay between cultural, social, and environmental influences. Future studies should explore these dynamics in greater depth, using qualitative methods or longitudinal designs to disentangle the relationships between prosocial participation and risk factors in the Colombian context.

Like the risk factors in the family domain, the factorial structure of the individual and peer group domains presents several possible solutions. Residual errors, particularly in items related to drug use, such as the early onset of drug use factor, are observed. However, in this case, residual errors occur with items from other factors, including sensation seeking, making modification of the indices unviable. We propose that these covariances of items related to drug use may be due to the perception that adolescents have regarding drugs that are considered legal for adults but are also used by adolescents in a normalized manner, such as marijuana. These perceptions may be influenced by cultural changes that currently tend towards legalization. It’s important to note that similar results have been observed in other factorial studies of CTC-YS regarding factors related to the use of legal drugs (De Oliveira Corrêa et al., 2022).

We consider the CTC-YS to be suitable for assessing risk and protective factors in Colombian adolescents, but the results of this study should not be considered definitive for family, individual, and peer group risk factors. These factors need to be thoroughly reviewed, as suggested by Batista-Foguet et al. (2004), “it is difficult for CFA models to fit the data in the first contrast” (p. 25), and a diagnosis should be made to evaluate the model and find ways to improve it.

This study has several limitations. First, the risk factors in the family, individual, and peer group domains did not reach optimal goodness-of-fit indices, and analyzing the items with the highest error may improve the indicators. Second, other necessary analyses were not performed, such as gender and age invariance, cutoff points for risk and protective factors, and others validity criteria analysis. Third, a cross-sectional measurement and a single sample were used, limiting the conclusions about the results. Fourth, three factors were not included: transition and mobility (community), gang membership, and social skills (individual and peer groups), which affected the domain results. Future studies can improve and integrate these aspects and address the limitations of this study.

Finally, while the survey was administered online and anonymously to minimize observation bias and promote honest responses, the presence of researchers to assist with questions may still have influenced participant behavior. However, strict measures were taken to ensure participant comfort and freedom, including preventing teachers or school staff from being present during survey completion. Additionally, researchers refrained from walking around the room while the adolescents answered the survey, intervening only when clarification was requested. These procedures aimed to create a neutral environment and reduce the likelihood of socially desirable responses.

Future research could further mitigate potential biases by exploring fully unsupervised methods, such as allowing adolescents to complete surveys in private settings or outside of the classroom environment.

## Conclusion

The analysis of the factorial structure of the CTC-YS survey in Colombian adolescents revealed its potential as an adequate tool for assessing risk and protective factors in adolescents. Although the overall factorial structure of the instrument aligns with the original model, significant limitations are evident in certain domains, particularly in the family and individual/group peer risk factors, which did not reach the expected goodness-of-fit indices. Careful cultural adaptation, thorough item review, and other psychometric analyses are necessary to improve the validity and applicability of the CTC-YS in Colombia.

## Data Availability

The data that support the findings of this study are available with request to the corresponding author.
